# Health Benefits of Mediterranean Diet

**DOI:** 10.3390/nu11081802

**Published:** 2019-08-05

**Authors:** Daniela Martini

**Affiliations:** Human Nutrition Unit, Department of Veterinary Science, University of Parma, 43125 Parma, Italy

Growing evidence shows that a dietary pattern inspired by Mediterranean Diet (MD) principles is associated with numerous health benefits [1,2]. A Mediterranean-type diet has been demonstrated to exert a preventive effect toward cardiovascular diseases, in both Mediterranean and non-Mediterranean populations [1,3]. These properties may in part depend on the positive action on the cardiometabolic risk [4,5], by decreasing the risk of diabetes and metabolic-related conditions [6,7,8]. There is also evidence of a potential role of the Mediterranean diet in preventing certain cancers [9]. Finally, a new field of research has showed that a higher adherence to the Mediterranean diet is associated with a lower risk of mental disorders, including cognitive decline and depression [10,11,12]. Overall, a better understanding of the key elements of this dietary pattern, the underlying mechanisms, and targets, are needed to corroborate current evidence and provide insights on new and potential outcomes.

The Special Issue “Health Benefits of Mediterranean Diet” was devoted to collect original research and reviews of literature concerning the Mediterranean diet and various health outcomes. New information has been added in this field by means of 16 articles, with nine original papers, six reviews/meta-analysis and one opinion.

A widely considered aspect was the evaluation of the adherence to the MD in different target populations. An Italian study found a poor adherence in 16.7% of a group of 669 subjects (6–16 years) attending five schools from the North Italy, with poor adherence more frequent in primary than in secondary schools [13].

Among two other studies from Spanish groups of research, the first investigated the relationship between adherence to MD, physical activity, self-concept (i.e., the collection of beliefs about oneself), and other sociodemographic factors in a group of Spanish university students. Results showed that MD adherence, measured by means of the Mediterranean Diet Quality Index (KIDMED), was associated with academic and physical self-concept [14]. 

In the second one, Muros and Zabala investigated the adherence to MD in two groups of cyclists and triathletes, finding a large proportion of the surveyed athletic population not meeting the MD guidelines, with particularly low adherence amongst men and cyclists [15].

The adherence to the MD was also investigated by the group of Chacon-Cuberos who evaluated the relationships between adherence to the MD and motivational climate in sport on a sample of university students from Spain and Romania [16]. Results showed a higher adherence in students from Spain compared to Romanians and observed that ego-oriented climates (measured as unequal recognition, member rivalry and punishment for mistakes) are linked to a better adherence to the MD, especially due to the importance of following a proper diet in sport contexts.

Besides measuring the adherence to MD in different contexts, it is also interesting to identify the perceived barriers to follow the MD, which might lead to low MD adherence. In this scenario, Kretowicz and colleagues considered women of childbearing age and identified five barriers and enablers (Mediterranean diet features, perceived benefits, existing dietary behavior and knowledge, practical factors, and information source) that should be considered in the design and development of an intervention to effectively promote and encourage adherence to the MD [17]. 

As already mentioned, a high adherence to the MD has been extensively associated with a number of health outcomes, and several manuscripts of this Special Issue explored this association. Firstly, two observational studies by Godos and colleagues [18,19] showed a linear association between the overall quality of life and adherence to the MD score in a cohort of over 2000 Italian adults. Authors also evaluate whether subjects found a better sleep quality by following MD, either toward direct effect on health or indirect effects through improvement of weight status.

The role of MD on body weight and other outcomes has been also investigated by an Italian group who observed that a high MD score was associated with lower values of plasma lipids and glycated hemoglobin, blood pressure and body mass index in people with type-2 diabetes [20], thus supporting the MD as a suitable model also in these subjects. The association between MD and obesity has been also considered and discussed in other two reviews included in this Special issue. 

Firstly, Castro-Barquero et al. reviewed the evidence on the relationship between the polyphenol intake in the frame of the MD (e.g., from extra-virgin olive oil, nuts and legumes) and obesity [21]. Findings evidenced that, despite the intake of some specific polyphenols has been associated with body weight improvements, there is no strong evidence of an association between polyphenols intake and lowering of body adiposity. 

A second review was proposed by D’Innocenzo and coworkers who highlighted the importance of public policy measures to make a healthy diet easily accessible and affordable. [22] This advice has been deepen as for general population as for target groups (e.g., children and adolescents), in order to tackle obesity epidemic by considering that “Diet as not just a food model, but also as the most appropriate regime for disease prevention, a sort of complete lifestyle plan for the pursuit of healthcare sustainability”.

The adherence to the MD has been also associated to inflammation in an investigation performed within the HELENA study [23]. Results evidenced a counteracting effect of stress on inflammatory biomarkers with high MD adherence, with stress being a significant independent negative predictor of a healthy dietary pattern.

Lastly, the study by Del Bo’ and colleagues systematically reviewed the human intervention studies evaluating the impact of Mediterranean diet on markers of DNA damage, reporting a reduction in the levels of 8-hydroxy-2′–deoxyguanosine and a modulation of DNA repair gene expression and telomere length [24].

The Special Issue also included other aspects relating to the MD, such as serving size and translation of the MD to other diets. Firstly, D’Alessandro and coworkers performed a systematic review of dose-response meta-analyses of prospective studies, which evaluated the association between the intake of food groups belonging to a variant of the Modern Mediterranean Diet Pyramid and the risk of CVD [25]. Among the different aspects worth to be studied, the serving sizes of the foods seems to have a key role in order to obtain a protective or a not detrimental effect toward selected diseases. This supports the idea that a throughout definition of MD must consider not only the types of food but also their amounts and frequency of consumption. Being the efficacy and feasibility of MD for the management of chronic diseases not been extensively evaluated in non-Mediterranean settings, the paper by George and coworkers [26] increased knowledge about potential strategies for translating the traditional MD into a non-Mediterranean setting.

A different shade of evidence has been added by the work of Biagi et al., a systematic review investigating the effect of the MD during pregnancy on birth outcome [27]. Despite authors concluded that data were insufficient and further randomized control trials are needed to draw clear conclusions, growing evidence seem to suggest a beneficial effect of the Mediterranean diet during pregnancy on children’s health.

One of the main challenges when trying to investigate the role of the MD on health is related to the accurate assessment of exposure to this dietary pattern. In this framework, the manuscript by Jin et al. focused on metabolomics and gut microbiota role for evaluating the MD effects by summarizing the current evidence from observational and clinical trials [28].

Overall, the studies included in the Special issue provide new insights on the protective effect of MD on health, although many authors reported the need of performing further investigations to confirm these effects.
